# Inter-Reader Variability in the Classification of Pneumoconiosis Using the International Labour Organization International Classification System on Chest Radiographs of Artisanal and Small-Scale Gold Miners Attending a Screening Program in Kwe Kwe District, Zimbabwe

**DOI:** 10.3390/ijerph23081060

**Published:** 2026-08-15

**Authors:** Dingani Moyo, Anil Chuturgoon, Eduardo Mello De Capitani, Rajen N. Naidoo

**Affiliations:** 1 School of Medicine, College of Health Sciences, University of Kwa-Zulu Natal, Durban 4041, South Africa; chutur@ukzn.ac.za (A.C.); naidoon@ukzn.ac.za (R.N.N.); 2 School of Public Health, Faculty of Health Sciences, University of the Witwatersrand, Johannesburg 2000, South Africa; 3 Department of Community Medicine, Faculty of Medicine, National University of Science and Technology, Bulawayo P.O. Box AC 939, Zimbabwe; 4 Department of Internal Medicine, School of Medicine, State University of Campinas, Campinas 13083-970, Brazil; capitani@unicamp.br

**Keywords:** inter-reader variation, chest radiography, artisanal miners, ILO classification

## Abstract

**Highlights:**

**Public health relevance—How does this work relate to a public health issue?**

- This study demonstrates that substantial inter-reader variability persists in the radiographic classification of pneumoconiosis, particularly for early-stage disease.
- By providing evidence from artisanal and small-scale gold miners in Zimbabwe, these findings inform occupational health surveillance strategies in low- and middle-income countries (LMICs) and support efforts to improve the accuracy of pneumoconiosis screening and disease surveillance.

**Public health significance—Why is this work of significance to public health?**

- Reliable radiographic classification is fundamental to effective pneumoconiosis surveillance and prevention programs. Improving the International Labour Organization (ILO) category classification in early pneumoconiosis can facilitate timely removal of exposed miners, reducing disease progression.
- The findings provide evidence to strengthen occupational health capacity in LMICs by supporting targeted reader capacity development, standardized quality assurance, and consensus chest radiograph reading strategies, thereby improving the accuracy of pneumoconiosis surveillance among miners.

**Public health implications—What are the key implications or messages for practitioners, policy makers, and/or researchers in public health?**

- Healthcare practitioners and radiologists should undertake regular continuing professional development and competency-based training in the application of the ILO International Classification of Radiographs of Pneumoconioses. Training should place particular emphasis on improving the recognition and classification of borderline radiographic abnormalities, especially ILO Category 1, where the greatest inter-reader variability occurs.
- Policy makers, occupational health authorities, and researchers should develop and implement national standards and policy frameworks for pneumoconiosis surveillance in exposed workers, particularly in the artisanal and small-scale mining sector. These frameworks should mandate regular medical surveillance using standardized ILO classification.

**Abstract:**

Pneumoconiosis remains a major problem globally with a much higher burden in low- and middle-income countries (LMICs). There is increased inter-reader variability in the classification of pneumoconiosis in most LMICs. Despite the use of the International Labour Organization (ILO) International Classification System that standardizes reporting of pneumoconiosis on chest radiographs, inter-reader variability persists. We aimed to assess inter-reader concordance in classifying pneumoconiosis using the ILO International Classification System among artisanal and small-scale gold miners (ASGMs) in Zimbabwe. A retrospective cross-sectional study was conducted using 576 eligible chest radiographs (dicom format). Overall agreement for binary classification was moderate (κ = 0.522, 95% CI: 0.475–0.569), with pairwise agreement ranging from moderate to substantial (κ = 0.464, 95% CI: 0.389–0.538 to κ = 0.681, 95% CI: 0.602–0.761). Agreement was consistently higher for radiographs not suggestive of pneumoconiosis, with agreement ranging from 85.9% to 94.4%. Across the four ILO profusion categories, substantial inter-reader agreement was observed, with linear weighted kappa ranging from (κ = 0.554, 95% CI: 0.483–0.625 to κ = 0.668, 95% CI: 0.598–0.739) and quadratic weighted kappa values ranging from (κ = 0.675, 95% CI: 0.604–0.747 to κ = 0.755, 95% CI: 0.685–0.826). Cross-tabulation analyses demonstrated that disagreement occurred predominantly between adjacent profusion categories. Category-specific analyses showed the highest concordance for Categories 0 and 3, while Category 1 demonstrated the poorest agreement, with κ values ranging from (κ = 0.091, 95% CI: 0.004–0.178 to κ = 0.216, 95% CI: 0.067–0.365). Inter-reader agreement was slightly higher among chest radiographs of ASGMs with previous tuberculosis treatment, with κ values ranging from (κ = 0.572, 95% CI: 0.360–0.784 to κ = 0.743, 95% CI: 0.565–0.921) than among those without with κ values ranging from (κ = 0.376, 95%C1: 0.291–0.460 to κ = 0.602, 95% C1: 0.498–0.706). These findings reflect substantial variability in classifying early or borderline pneumoconiotic changes. These findings highlight variability in radiographic interpretation and demonstrate the need for enhanced training, quality control, and consensus reading approaches in ASGMs’ screening programs.

## 1. Introduction

Globally, silicosis remains one of the most prevalent and significant pneumoconioses, with an estimated 234,160 cases reported in 2021 [1]. Pneumoconiosis is an irreversible chronic fibrotic lung disease caused by inhalation of mineral dusts such as silica dust, asbestos dust, and coal dust, among many other important dusts. Silicosis results from inhalation of silica dust. Although the incidence of silicosis has declined in many developed countries, a recent resurgence has been documented in Australia and the United Kingdom [2]. In contrast, low- and middle-income countries (LMICs) are experiencing a growing epidemic of silicosis, particularly among artisanal and small-scale gold miners (ASGMs), with the burden being especially pronounced in Africa [3,4,5,6]. Furthermore, southern Africa is currently experiencing a triple epidemic of silicosis, human immunodeficiency virus (HIV) infection, and tuberculosis (TB). Zimbabwe has a high prevalence of silicosis, affecting up to 20% of ASGMs [3,7].

Despite this high prevalence, the diagnosis of silicosis in LMICs remains challenging, largely due to the high burden of tuberculosis, which often presents with similar radiological features [4,8,9]. This overlap frequently complicates the accurate diagnosis of silicosis [10]. Furthermore, the classification of silicosis for epidemiological purposes remains a major challenge in many developing countries. To promote consistency and standardization in the reporting of pneumoconioses, the International Labour Organization (ILO) developed a standardized system for classifying chest radiographs [11]. The ILO International Classification of Radiographs of Pneumoconioses provides a systematic method for describing and recording radiographic abnormalities caused by the inhalation of dust. It also provides a set of standard images covering the whole range of profusion categories that must be used for comparison with the image being interpreted, aimed at reducing readers’ subjectivity. The revised 2022 edition of the ILO classification system introduced fully digital standard images, replacing the digitized analog images used in the 2011 edition [11].

Despite its widespread use and utility, significant inter-reader variability persists when applying the ILO classification system. Accurate and consistent classification of chest radiographs of pneumoconioses is critical for epidemiological studies, screening programs, and surveillance activities aimed at controlling pneumoconiosis [12,13,14]. Although the ILO Classification was not designed for computing compensation, several countries use the ILO classification for evaluating the degree of impairment and compensation. It is important for readers to have consistency in reporting chest radiographs for pneumoconiosis to accurately characterize the burden of pneumoconiosis. While the ILO International Classification System provides a standardized framework for interpreting chest radiographs, evidence on inter-reader agreement in artisanal and small-scale gold mining populations, particularly in low- and middle-income countries, remains limited. Therefore, the objective of this study was to examine the degree of inter-reader agreement in classifying radiographs for pneumoconiosis using reading results of three specialist occupational physicians.

## 2. Materials and Methods

A retrospective cross-sectional review of chest radiographs of ASGMs who attended an outreach screening program in Kwe Kwe District in Zimbabwe was conducted by three specialist occupational physicians from three different countries: Zimbabwe, South Africa, and Brazil. The chest radiographs were from ASGMs who were actively involved in mining and exposed to silica dust. The outreach activity was done at or close to the mining sites where ASGMs attended during the start or end of their shifts or their work breaks.

### 2.1. Study Sample

The study sample consisted of 576 eligible chest radiographs in dicom format that were read by all three specialist physicians from a sample of 693 ASGMs who voluntarily attended an outreach screening.

**Figure 1:**
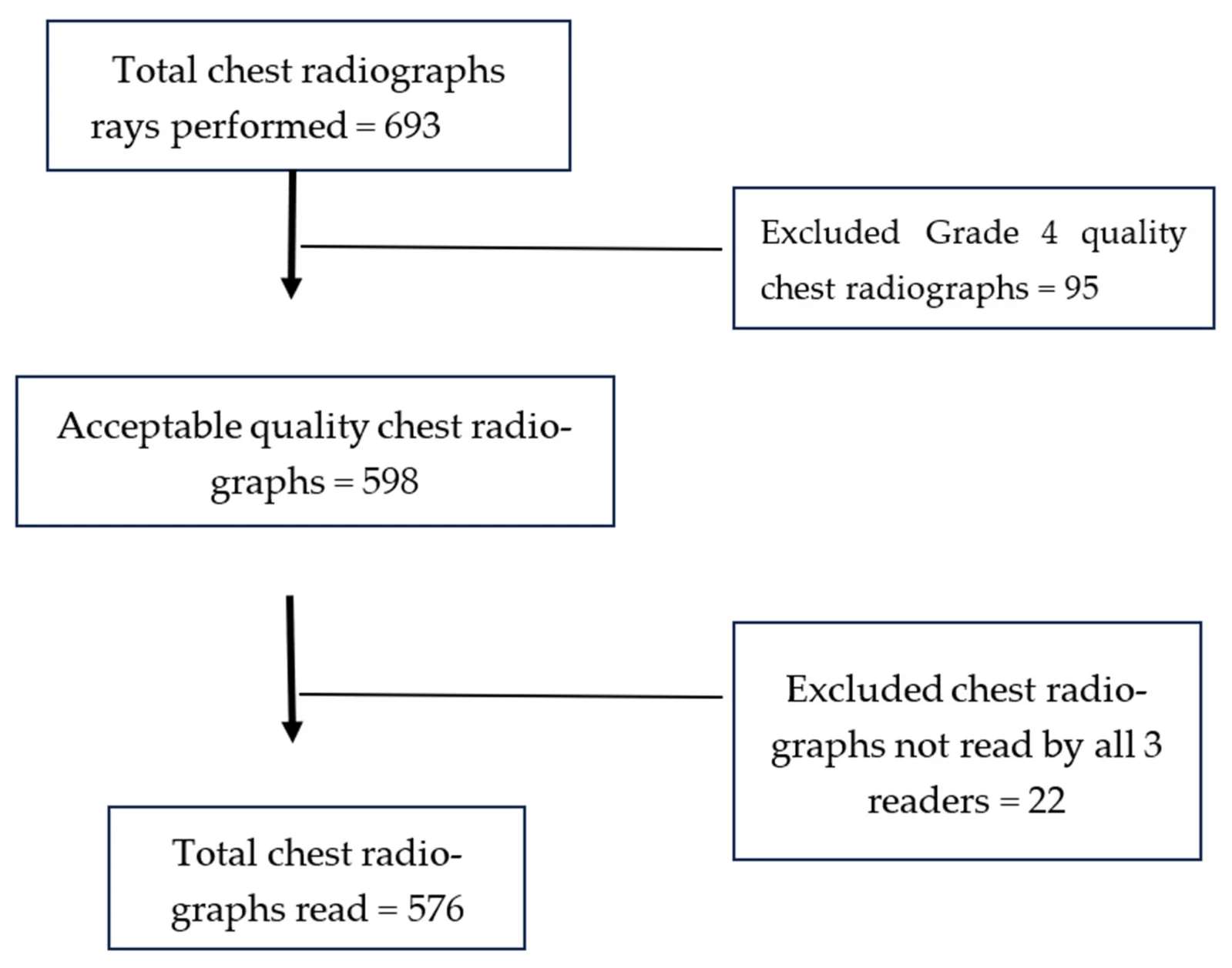
Flow diagram for sample selection.

From a sample of 693 chest radiographs, 576 met the eligibility criteria after accounting for chest radiographs that were classified as ILO quality grade 4 (unacceptable for classification purposes) or were not read by one or two of the occupational physicians due to missing dicom files or challenges with accessing the files. The flow diagram is shown in Figure 1.

### 2.2. Characteristics of the Readers

All the readers had at least 15 years of experience in Occupational Medicine and use of the ILO system for the classification of radiographs of pneumoconioses. One of the readers was a National Institute for Occupational Safety and Health (NIOSH) certified B Reader.

### 2.3. Classification of Chest Radiographs

The main rating of interest was profusion scored from 0/− to 3/+. Chest radiographs were read and classified independently by the three readers. The readers knew that the chest radiographs were of ASGMs but were blinded to clinical information, exposure history, and each other’s readings. All the readings were conducted independently using the ILO 2022 digital standard images for comparison purposes [11]. Each reader received five folders with the chest radiographs in dicom format and read them according to their preferred order. Chest radiograph interpretation was performed by the readers in their respective countries using one of the following medical monitors and software: (i) Pixviewer software (Pixmeo SARL, Geneva, Switzerland), x-view software, on a calibrated 3-megapixel EIZO diagnostic display monitor [15], (ii) Weasis Medical Viewer 4.7.0 and the monitor was a Dell P2715Q 4K IPS Ultra HD (3840 × 2160 resolution) [16], and (iii) ProLite T2236MSC-B2 software and a 22” 10 point touch monitor with edge-to-edge glass and AMVA panel [17]. The chest radiographs were read in a dedicated reading environment under controlled ambient lighting conditions to minimize screen glare. Readers were able to adjust image display parameters, including magnification, width, and pan functions, among others, as needed to facilitate interpretation.

The threshold for chest radiograph findings consistent with pneumoconiosis was defined as an ILO profusion category of ≥1/0, while chest radiograph findings not consistent with pneumoconiosis were defined as ≤0/1. The ILO classification system assesses the profusion of small opacities using four major categories: 0, 1, 2, and 3. Category 0 represents chest radiographs with findings that are not consistent with pneumoconiosis, while categories 1, 2, and 3 represent chest radiographs with profusion findings consistent with pneumoconiosis in increasing severity. Each major category is further subdivided into three subcategories, yielding 12 profusion sub-categories: 0/−, 0/0, 0/1; 1/0, 1/1, 1/2; 2/1, 2/2, 2/3; and 3/2, 3/3, 3/+. The ILO main category 0 consists of three subcategories 0/−, 0/0, and 0/1, while the ILO main category 1 consists of 1/1, 1/2; 2/1. The categories 0/1 and 1/0 represent borderline classifications and are particularly important when determining the presence or absence of chest radiograph findings consistent with pneumoconiosis. A classification of 0/1 indicates that the reader’s primary assessment was category 0 (findings not consistent with pneumoconiosis), but category 1 was also considered as a possible alternative. Thus, the radiograph was ultimately interpreted as not consistent with pneumoconiosis, although some features suggestive of pneumoconiosis were noted by the reader. Conversely, a classification of 1/0 indicates that the reader’s primary assessment was category 1, with category 0 considered as the alternative interpretation. In this case, the radiograph was classified as consistent with pneumoconiosis, although the possibility of a radiograph not consistent with pneumoconiosis was also acknowledged.

### 2.4. Consensus Reading Approach

The consensus reading for the ILO profusion subcategory was determined when at least two readers independently recorded the identical ILO classification subcategory and accompanying symbols. In cases where all three readers assigned different ILO subcategories within the same major profusion category, the median ILO subcategory was designated as the consensus classification. When the three readings spanned different major profusion categories, the classification assigned by the NIOSH-certified B Reader was used as the consensus rating. This was considered the most appropriate reference standard because the B Reader was the only panel member with formal NIOSH B Reader certification and had the greatest experience in applying the ILO classification system.

### 2.5. Inclusion and Exclusion Criteria

All chest radiographs that were read by all three readers and were classified as grade 1–3 quality (acceptable quality) were included in the study. All images that were not read by any one or two of the three readers were excluded from the study. Furthermore, chest radiographs that were either classified as Grade 4 quality (unacceptable for classification) or were not read by one reader due to missed or corrupted chest radiograph files were excluded.

### 2.6. Ethical Approval

Permission to conduct the study was obtained from the Secretary for Health and Child Care, Ministry of Health and Child Care, Zimbabwe, and from the Zimbabwe Artisanal and Small-Scale Miners’ Association. Ethics approval was obtained from the Medical Research Council of Zimbabwe (MRCZ) (Approval number: MRCZ/A/3133).

### 2.7. Statistical Analysis

Data from the three readers were analyzed using International Business Machines Statistical Package for the Social Sciences (IBM^®^ SPSS^®^) Statistics version 27.0.1.0 [18]. Inter-reader variation was assessed using conditional probabilities and Cohen’s kappa [16] to quantify consistency across readers for findings consistent or not consistent with pneumoconiosis. To account for the ordinal nature of the ILO classification system of chest radiographs, Linear weighted kappa and quadratic weighted kappa were used. Data were inspected for completeness and coding consistency, with missing and invalid readings being excluded in the final analysis. Continuous variables such as age, age at start of artisanal and small-scale gold mining work, and years in artisanal and small-scale mining were converted to numeric formats, and descriptive statistics like mean and standard deviation were calculated. Inter-reader agreement among three readers was assessed for binary classification (suggestive of pneumoconiosis and not suggestive of pneumoconiosis) using kappa statistics. Kappa values were interpreted according to standard benchmarks (≤0.20 = poor, 0.21–0.40 = fair, 0.41–0.60 = moderate, 0.61–0.80 = substantial, ≥0.81 = almost perfect agreement) [19].

## 3. Results

Table 1 presents the demographic and clinical characteristics of the study population. A total of 576 chest radiographs from ASGMs were analyzed. The mean age of the ASGMs was 35.3 years (SD 11.6), while the mean working experience was 25.3 years (SD = 9.7). The cohort was predominantly male (88.0%). Most participants (79.7%) completed secondary education, whereas 15.5% had attained primary education. Radiographic findings consistent with pneumoconiosis were observed in 18.4% of the radiographs.

The distribution of chest radiographic classifications varied across readers, with all readers classifying most of the chest radiographs as ILO Category 0, as shown in Table 2. Reader 1 classified a substantially higher proportion of radiographs as Category 1, 95 (16.5%), and Category 2, 83 (14.4%), compared with Readers 2 and 3, who assigned fewer readings to these categories. In contrast, Reader 2 recorded the highest proportion of Category 3 classifications, 44 (7.6%). These findings suggest greater variability in the assignment of intermediate ILO categories than in the identification of chest radiographs not suggestive of pneumoconiosis.

Inter-reader agreement among the three readers varied from moderate to substantial across the three reader-pairs, as shown in Table 3. Despite the observed agreement being similar between Reader 1 and Reader 2 (79.3%) and between Reader 1 and Reader 3 (79.2%), the corresponding Cohen’s kappa coefficients indicated only moderate agreement (k = 0.485, 95% CI: 0.410–0.561 and k = 0.464, 95% CI: 0.389–0.538), respectively. Furthermore, for the same reading pairs, positive agreement (chest radiographs suggestive of pneumoconiosis) was modest for these comparisons at 61.2% and 57.7%, respectively, whereas negative agreement (chest radiographs not suggestive of pneumoconiosis) was consistently high (85.9% and 86.2%). The highest level of concordance was observed between Reader 2 and Reader 3, with an observed agreement of 90.8% and substantial agreement (k = 0.681, 95% CI: 0.602–0.761). Reader 2 and Reader 3 also demonstrated the greatest positive agreement at 73.6% and negative agreement at 94.4%, indicating improved consistency in identifying both positive and negative radiographic findings. Overall, inter-reader agreement was consistently higher for classifying negative than positive findings, as depicted by uniformly greater negative agreement across all reader-pairs.

Inter-reader agreement across the main ILO classification Categories (0–3) ranged from mild to moderate, with unweighted kappa values ranging from (k = 0.378, 95% CI: 0.314–0.442) to (k = 0.539, 95% CI: 0.464–0.614), as shown in Table 4. Inter-reader concordance improved when accounting for the ordinal nature of ILO categories, with linear weighted kappa values ranging from (k = 0.554, CI: 0.483–0.625) to (k = 0.668, 95% CI: 0.598–0.739), while quadratic weighted kappa values demonstrated substantial agreement across all reading pairs. The highest level of agreement was observed between Readers 2 and 3 (k = 0.755, 95% CI: 0.685–0.826), indicating greater consistency in assigning ILO categories, while agreement involving Reader 1 and Reader 3 was comparatively lower (k = 0.675; 95% CI: 0.604–0.747).

Pairwise comparison of ILO classifications between Reader 1 and Reader 2 demonstrated the highest agreement for Category 0 and Category 3, as shown in Supplementary Table S1. In contrast, concordance was much lower for the intermediate categories, with only 10 Category 1 readings and Category 2 readings assigned concordantly. Most variability involved classification between adjacent categories, especially Categories 1 and 2, indicating that reader variability was greatest in the interpretation of borderline radiographic abnormalities rather than those at the extremes, i.e., Category 0 and Category 3.

Cross-tabulation for Reader 1 and Reader 3, as indicated in Supplementary Table S2, demonstrated the highest concordance at the extremes of the ILO classification scale, with 374 participants classified as Category 0 and 17 as Category 3 by both readers. Disagreement was most evident between adjacent categories, particularly where 78 participants were classified as Category 1 by Reader 1 but Category 0 by Reader 3, and 35 participants were classified as Category 2 by Reader 1 but Category 0 by Reader 3. Inter-reader concordance for Category 2 was comparatively lower, with 29 participants receiving identical classifications. Overall, the pattern of disagreement was predominantly confined to neighboring ILO profusion categories.

Cross-tabulation for Reader 2 and Reader 3 showed strong concordance for Category 0 with 449 chest radiographs and Category 3 with 22 radiographs, Supplementary Table S3. Inter-reader non-concordance was primarily confined to adjacent ILO profusion categories, including 17 radiographs classified as Category 1 by Reader 2 but classified as Category 0 by Reader 3, and 15 participants classified as Category 2 by Reader 2 but Category 0 by Reader 3. Agreement for Category 2 was moderate, with 15 participants receiving identical classifications. Overall, the pattern of disagreement was characterized by minor shifts between neighboring profusion categories, with relatively few large discrepancies, consistent with good inter-reader reliability in ordinal ILO classification.

Table 5 depicts category-specific kappas and conditional probabilities. Concordance between the reader-pairs was highest for the extreme ILO categories 0 and 3 where Category 0 had high conditional probabilities (P(B|A):0.953–0.982; P(A|B): 0.768–0.922) and moderate to substantial category specific kappa values ranging from (k = 0.464, 95% CI: 0.389–0.538) to (k = 0.681, 95% CI: 0.602–0.761). There was almost perfect agreement for category 3, where the strongest agreement occurred between Reader 1 and Reader 3, with a conditional probability of (P(A|B) = 0.957) and kappa values of (k = 0.638, 95% CI: 0.502–0.773). Across all reader-pairs, Category 1 showed poor to fair agreement across all the reading pairs ranging from (k = 0.091, 95% CI:0.004–0.178)–(k = 0.216, 95 CI:0.067–0.365) while there was fair to moderate agreement for Category 2 (k = 0.378, 95% CI: 0.229–0.527) to (k = 0.455, 95% CI: 0.342–0.569). Conditional probabilities P(B|A) and P(A|B) were consistently low across all reader-pairs for Category 1 and Category 2. Overall, the results show strong concordance for extreme ILO categories, 0 and 3, while Category 1 exhibited the worst concordance, followed by Category 2.

Inter-reader agreement was generally higher among chest radiographs of ASGMs with a history of TB than among those without, as shown in Table 6. Observed agreement ranged from 80.7% to 87.7%, with moderate to substantial agreement (k = 0.572, 95% CI: 0.360–0.784) to (k = 0.743, 95% CI: 0.565–0.921). The highest level of agreement was observed between Readers 2 and 3 (κ = 0.743, 95% CI: 0.565–0.921), accompanied by high positive agreement 89.9% and negative agreement 84.4%. For chest radiographs where there was no previous history of TB, observed agreement ranged from 78.9% to 91.1%, with, however, low kappa values (κ = 0.376–0.602), depicting fair to substantial agreement.

## 4. Discussion

Overall, inter-reader concordance was moderate for classifying chest radiographs as suggestive or not suggestive of pneumoconiosis (unweighted κ = 0.522, 95% CI: 0.475–0.569), with pairwise binary agreement ranging from moderate to substantial. Agreement was consistently higher for radiographs not suggestive of pneumoconiosis, as reflected by greater negative agreement across all reader-pairs. Unweighted kappa values for classification across ILO categories ranged from mild to moderate, whereas linear and quadratic weighted kappas demonstrated substantial agreement after accounting for the ordinal nature of the ILO scale. Cross-tabulations showed the greatest variability in adjacent and borderline ILO categories, particularly Category 1, followed by Category 2, while concordance was highest at the extremes (Categories 0 and 3). Category-specific kappa and conditional probability analyses similarly demonstrated strong agreement for Categories 0 and 3 but the poorest agreement for Category 1.

We found an overall substantial agreement (quadratic weighted k = 0.710; CI: 0.644–0.763) across the three readers. Our findings contrast those of Laney et al. (2011), where B readers who rated 172 chest radiographs showed moderate agreement (weighted k = 0.58; 95% CI; 0.54–0.62) [20]. In addition, our study findings further contrast those of Impivaara et al. (1995), who demonstrated moderate inter-reader agreement (k = 0.48; 95% CI: 0.46–0.49) [21]. Our study sharply contrasts with another study by Halldin, CN et al. (2017) where there was minimal agreement (weighted k = 0.235; 95% CI 0.22–0.25) among A and B readers on the profusion of small opacities [22]. Furthermore, our study findings showed a better agreement than Franzblau, A et al. (2018)’s study, which revealed a fair agreement (k = 0.37; 95% CI: 0.30−0.44) on parenchymal abnormalities and even poorer agreements for other outcomes such as large opacities and pleural findings [23]. Our study findings contrast greatly with Attified, MD. et al. (1986)’s findings that demonstrated a large inter-reader variability on a sample of 14,883 chest radiographs [24].

We found strong concordance for extreme ILO categories, 0 and 3 with category 0 having the highest conditional probabilities (P(B|A):0.953–0.982; P(A|B): 0.768–0.922) and moderate to substantial category specific linear weighted kappa values ranging from (k = 0.464, 95% CI: 0.389–0.538) to (k = 0.681, 95% CI: 0.602–0.761). Similar findings were obtained by Welch LS et al. (1998), who demonstrated higher concordance for normal chest radiographs than those that showed disease [25].

In our study, ILO Category 1 showed the poorest concordance, with linear weighted κ values ranging from poor (κ = 0.091, 95% CI: 0.004–0.067) to fair (κ = 0.216, 95% CI: 0.067–0.365), indicating substantial inconsistency in classification. Cross-tabulation further showed that discordance was largely confined to Categories 1 and 2, while Categories 0 and 3 demonstrated consistently high concordance. Similar findings were reported by Musch et al. (1985), who observed significant discrepancies in distinguishing between major profusion Categories 0 and 1, reflecting the challenge of classifying early pneumoconiosis, when removal from silica exposure is most beneficial [13]. Our findings are also consistent with Laney et al. (2011), who reported fair inter-reader agreement for dichotomized small opacity profusion (κ = 0.39, 95% CI: 0.28–0.49) [26], and Marques et al. (1989), who found considerable inter-reader variability in interpreting early radiological changes of silicosis [27].

As expected, Category 3 demonstrated substantial to almost perfect agreement, with linear κ values ranging from (κ = 0.53, 95% CI: 0.350–0.657) to (κ = 0.845, 95% CI: 0.723–0.967) across the three readers. As the most severe and readily recognizable form on chest radiographs, this finding was anticipated and is consistent with Mikulski et al., who reported the highest concordance for films with the greatest profusion scores [28]. Overall, these findings suggest that although inter-reader agreement was moderate, considerable variability remained in the classification of Category 1.

The observed inter-reader variability has important implications for compensation systems that rely on chest radiograph classification to determine eligibility for pneumoconiosis benefits. Moderate overall agreement and particularly poor concordance for Category 1 profusion suggest that workers with early pneumoconiosis may be classified differently depending on the reader. Such variability may result in inconsistent compensation decisions. However, it is important to note that the ILO classification was not designed for compensation purposes.

Our study has several strengths. This study is among the first to comprehensively evaluate inter-reader agreement using the ILO International Classification among ASGMs in Zimbabwe. It is further strengthened by the inclusion of a large sample size of 576 chest radiographs. Three experienced readers from different countries and continents, including a NIOSH-certified B Reader, strengthen the robustness and relevance of the study. By combining multiple complementary agreement metrics with category-specific analyses, it demonstrates that diagnostic variability is concentrated in ILO Category 1 (early pneumoconiosis), while agreement remains high for normal radiographs and advanced disease. We used Cohen’s kappa, linear weighted kappa, quadratic weighted kappa, confidence intervals, and conditional probabilities to provide for an exhaustive and critical analysis of inter-reader concordance. The analysis of concordance across the ILO-specific categories using both linear weighted and quadratic weighted kappa is an added strength of our study as it demonstrates the specific concordance across the ordinal ILO categories and aids in targeted interventions in capacity development for chest radiograph readers. These findings provide practical evidence to guide reader training, quality assurance, and occupational health surveillance in mineral dust-exposed populations in low- and middle-income countries.

Our study was not without limitations. First, it evaluated inter-reader variability only for ILO profusion classification and did not assess agreement for other radiographic parameters, including the shape and size of small opacities, large opacities, pleural abnormalities, and chest radiograph quality. Second, chest radiographs were obtained during outreach screening, and applying the ILO classification system assumed occupational silica dust exposure among all ASGMs. Despite these limitations, our findings confirm the inter-reader variability reported in previous studies. Future research should evaluate agreement across the full range of ILO radiographic parameters in ASGMs. Nevertheless, this is one of the first large studies to investigate inter-reader variability in chest radiograph interpretation among ASGMs.

## 5. Conclusions

Interpretation of chest radiographs for pneumoconiosis using the ILO Classification system showed moderate overall inter-reader agreement, improving to substantial concordance when the ordinal nature of the classification was considered. There was high consistency in identifying chest radiographs not suggestive of pneumoconiosis and those suggestive of advanced pneumoconiosis. The greatest inter-reader variability occurred in ILO Category 1, indicating considerable inconsistency in classification for this category. These findings underscore the need for standardized reader training, regular calibration exercises, and on-going quality assurance to strengthen pneumoconiosis surveillance, particularly in low- and middle-income countries with high mining activities.

## Figures and Tables

**Table 1:** Study population demographics and clinical characteristics.

Characteristic	Category	Frequency (N)	Proportion (%)
Total	-	576	-
Age (years)	Mean (SD)	35.3	SD = 11.6
Gender	Male	507	88.0
Years working as an ASGM	Mean	25.3	SD = 9.7
Level of education	Primary	89	15.5
	Secondary	459	79.7
	Tertiary	15	2.6
	Unknown	13	2.3
Chest radiographs	Suggestive of pneumoconiosis (positive)	106	18.4
HIV status	Positive	8	1.4
Previous TB treatment (N = 573)	Yes	57	9.9
Current TB treatment (N = 571)	Yes	9	1.6%

**Table 2:** ILO category distributions per reader.

Reader	Category 0 (N, %)	Category 1 (N, %)	Category 2 (N, %)	Category 3 (N, %)
Reader 1	381 (66.1)	95 (16.5)	83 (14.4)	17 (3.0)
Reader 2	464 (80.6)	29 (5.0)	39 (6.8)	44 (7.6)
Reader 3	487 (84.5)	33 (5.7)	33 (5.7)	23 (4.0)

**Table 3:** Pairwise binary agreement for chest radiographs suggestive and not suggestive of pneumoconiosis.

Comparison	N	Observed Agreement (Po)	Unweighted Kappa (k)	95% CI	Positive Agreement (Ppos)	Negative Agreement (Pneg)	Interpretation
Reader 1 vs. Reader 2	576	0.793	0.485	0.410–0.561	0.612	0.859	Moderate
Reader 1 vs. Reader 3	576	0.792	0.464	0.389–0.538	0.577	0.862	Moderate
Reader 2 vs. Reader 3	576	0.908	0.681	0.602–0.761	0.736	0.944	Substantial
Overall Agreement (3 Readers)	576	0.831	0.522	0.475–0.631	0.569	0.890	Moderate

**Table 4:** Inter-reader agreement (unweighted versus weighted kappa) across the ordinal ILO classification categories (0–3).

Comparison	N	Observed Agreement (Po)	Unweighted Kappa (k) (95% CI)	Linear Weighted Kappa (k) (95% CI)	Quadratic Weighted Kappa (k) (95% CI)
Reader 1 vs. Reader 2	576	0.722	0.378 (0.314–0.442)	0.561 (0.498–0.623)	0.704 (0.644–0.764)
Reader 1 vs. Reader 3	576	0.753	0.416 (0.347–0.485)	0.554 (0.483–0.625)	0.675 (0.604–0.747)
Reader 2 vs. Reader 3	576	0.858	0.539 (0.464–0.614)	0.668 (0.598–0.739)	0.755 (0.685–0.826)
Overall Agreement (3 Readers)	576	0.778	0.426 (0.394–0.458)	0.586 (0.523–0.642)	0.710 (0.644–0.763)

**Table 5:** ILO classification category-specific kappas and conditional probabilities for the reader-pairs.

Comparison	ILO Cat	Agreed (Nii) ^ῐῐ^	Rated by A ^‡^ (Ni.) ^ῐ^	Rated by B ^‡‡^ (N.i)	^‡‡‡^ P(B|A)	^‡‡‡‡^ P(A|B)	Category-Specific Linear Kappa	95% CI
Reader 1 vs. Reader 2	0	363	381	464	0.953	0.782	0.485	0.410–0.561
	1	10	95	29	0.105	0.345	0.091	0.004–0.178
	2	27	83	39	0.325	0.692	0.386	0.272–0.500
	3	16	17	44	0.941	0.364	0.503	0.350–0.657
Reader 1 vs. Reader 3	0	374	381	487	0.982	0.768	0.464	0.389–0.538
	1	14	95	33	0.147	0.424	0.146	0.050–0.242
	2	29	83	33	0.349	0.879	0.455	0.342–0.569
	3	17	17	23	1.000	0.739	0.845	0.723–0.967
Reader 2 vs. Reader 3	0	449	464	487	0.968	0.922	0.681	0.602–0.761
	1	8	29	33	0.276	0.242	0.216	0.067–0.365
	2	15	39	33	0.385	0.455	0.378	0.229–0.527
	3	22	44	23	0.500	0.957	0.638	0.502–0.773

^‡^ A = This refers to Reader 1 in the Reader 1 vs. Reader 2 pair and Reader 1 vs. Reader 3 pair, and refers to Reader 2 in the Reader 2 vs. Reader 3 pair. ^‡‡^ B: This refers to Reader 2 in the Reader 1 vs. Reader 2 pair, and in the Reader 1 vs. Reader 3 pair, while it refers to Reader 3 in the Reader 2 vs. Reader 3 pair. (Ni.) ^ῐ^: It is the number of chest radiographs placed in each category by each reader. (Nii) ^ῐῐ^: It is the number of radiographs that were independently classified by both readers into the same ILO category. ^‡‡‡^ P(B|A): represents the probability that Reader B rated the chest radiograph in a specific category given that Reader A rated the subject in the same category. ^‡‡‡‡^ P(A|B): represents the probability that Reader A rated the chest radiograph in a specific category given that Reader B rated the subject in the same category.

**Table 6:** Influence of previous TB treatment on inter-reader agreement.

Previous TB	Comparison	Valid N	Observed Agreement (Po)	Kappa (k) (95% CI)	Positive Agreement (Ppos) ^¥¥^	Negative Agreement (Pneg) ^¥^	Interpretation
Yes (N = 57)	Reader 1 vs. Reader 2	57	0.825	0.603 (0.392–0.814)	0.872	0.722	Substantial
	Reader 1 vs. Reader 3	57	0.807	0.572 (0.360–0.784)	0.857	0.703	Moderate
	Reader 2 vs. Reader 3	57	0.877	0.743 (0.565–0.921)	0.899	0.844	Substantial
No (N = 516)	Reader 1 vs. Reader 2	516	0.789	0.406 (0.320–0.493)	0.524	0.864	Moderate
	Reader 1 vs. Reader 3	516	0.789	0.376 (0.291–0.460)	0.473	0.868	Fair
	Reader 2 vs. Reader 3	516	0.911	0.602 (0.498 to 0.706)	0.652	0.949	Substantial

^¥^ Negative Agreement (Pneg): Refers to agreement on chest radiographs that are not suggestive of pneumoconiosis. ^¥¥^ Positive Agreement (Ppos): Refers to agreement on chest radiographs that are suggestive of pneumoconiosis.

## Data Availability

The data presented in this study are available on request from the corresponding author. The data are not publicly available due to authorizations that may be required by the Ministry of Health and Child Care in Zimbabwe.

## Supplementary Materials

The following supporting information can be downloaded at: https://www.mdpi.com/article/10.3390/ijerph23081060/s1, Table S1: Reader 1 and Reader 2 ILO Category Distributions and Raw Pairwise Cross Tabulations, Table S2: Reader 1 and Reader 3 ILO Category Distributions and Raw Pairwise Cross Tabulations, Table S3: Reader 2 and Reader 3 ILO Category Distributions and Raw Pairwise Cross Tabulations.

## Abbreviations

The following abbreviations are used in this manuscript:

ASGM	Artisanal and Small-Scale Gold Miner
HIV	Human Immunodeficiency Virus
ILO	International Labour Organization
LMICs	Low- and Middle-Income Countries
MRCZ	Medical Research Council of Zimbabwe
NIOSH	National Institute of Occupational Safety and Health
TB	Tuberculosis
