# Supplementary material for: Inter-Reader Variability in the Classification of Pneumoconiosis Using the International Labour Organization International Classification System on Chest Radiographs of Artisanal and Small-Scale Gold Miners Attending a Screening Program in Kwe Kwe District, Zimbabwe
Source: Int J Environ Res Public Health. 2026 Aug 15;23(8):1060. doi: 10.3390/ijerph23081060 (PMC13513877; doi:10.3390/ijerph23081060)
Supplement: Supplementary file 1 [file ijerph-23-01060-s001.zip › ijerph-4372725-supplementary.pdf]

SUPPLEMENTARY MATERIAL

Supplementary Table S1: Reader 1 and Reader 2 ILO Category Distributions and Raw Pairwise Cross Tabulations

Reader 1 \ Reader 2	Category 0	Category 1	Category 2	Category 3
Category 0	363	13	4	1
Category 1	75	10	7	3
Category 2	26	6	27	24
Category 3	0	0	1	16

Supplementary Table S2: Reader 1 and Reader 3 ILO Category Distributions and Raw Pairwise Cross Tabulations

Reader 1 \ Reader 3	Category 0	Category 1	Category 2	Category 3
Category 0	374	6	1	0
Category 1	78	14	3	0
Category 2	35	13	29	6
Category 3	0	0	0	17

Supplementary Table S3: Reader 2 and Reader 3 ILO Category Distributions and Raw Pairwise Cross Tabulations

**Reader 2\ Reader 3**	**Category 0**	**Category 1**	**Category 2**	**Category 3**
**Category 0**	449	12	3	0
**Category 1**	17	8	4	0
**Category 2**	15	8	15	1
**Category 3**	6	5	11	22
